# When circulatory death does not come in time in potential organ donors

**DOI:** 10.1186/s13054-019-2443-4

**Published:** 2019-05-02

**Authors:** Angela Kotsopoulos, Nichon Jansen, Wilson Farid Abdo

**Affiliations:** 1 Department of Intensive Care, Elisabeth-TweeSteden Hospital, Hilvarenbeekseweg 60, 5022 GC Tilburg, the Netherlands; 2 The Dutch Transplant Foundation, Leiden, the Netherlands; 3 Department of Intensive Care, Radboud University Medical Center, Nijmegen, the Netherlands

To the Editor

A substantial proportion of potential donors do not arrest in time in controlled donation after circulatory death (cDCD). Patients with a short time to death are well described in previous studies in attempts to develop models to predict the time between treatment withdrawal and circulatory death. However, studies that aimed to describe the group that did not arrest within the predefined timeframe are lacking.

We analyzed nationwide data of all 143 patients who entered the cDCD program, but in whom organs were not procured due to delayed circulatory death, in a period of 36 months. Additionally, we compared our cohort with the cohorts of five published studies on prognostic models predicting time to death in cDCD donors (Additional file 1: Table S1).

The majority of patients were male; median age at death was 57 years. Brainstem reflexes were mostly present, and the median Glasgow Coma Scale (GCS) was 4.

We found a variability in delayed death across countries. The UK and Australian cohorts showed the highest survival, and the cohort from China, the shortest [1, 3–5]. Such variation could be due to differences in palliative care provided. Patients could be under profound sedation which may directly affect (limit) the time period until death and hence the applicability of predictive models. All studies showed a wide variation in time to death. The effect of age was conflicting. Two studies found that older age was significantly associated to a longer survival [1, 5]. Two cohorts demonstrated a high prevalence of an extensor or absent motor response and is from a neuroanatomical perspective probably a more sensitive predictor than the GCS [2, 3]. The presence of gag or cough reflex was a protective factor for cardiac arrest [5].

Death was predominantly the resultant of neurologic injury; however, none of the diagnoses was associated with time to death [2, 4, 5]. A standardization of diagnostic categories was lacking across the studies making comparison difficult.

The greatest strength of our analysis was the evaluation of consecutive patients minimizing selection bias. Additionally, this is the largest cohort of cDCD donors with delayed time to death. The main drawback was the missing data on physiological parameters.

Based on our analysis, we recommend that age and brainstem reflexes should at least be studied in future studies on multimodal prediction models on time to death. There is an important knowledge gap in the effect of palliative practice on time to death.

## Additional file

Additional file 1: **Table S1.** Baseline characteristics of cDCD donors with a delayed time to death after withdrawal of life-sustaining therapy. (PDF 257 kb)

## Abbreviations

cDCD: Controlled donation after cardiac death
GCS: Glasgow Coma Scale
