# Supplementary material for: When circulatory death does not come in time in potential organ donors
Source: Crit Care. 2019 May 2;23:154. doi: 10.1186/s13054-019-2443-4 (PMC6498617; doi:10.1186/s13054-019-2443-4)
Supplement: Supplementary file 1 — Table S1. Baseline characteristics of cDCD donors with a delayed time to death after withdrawal of life-sustaining therapy. (PDF 257 kb) [file 13054_2019_2443_MOESM1_ESM.pdf]

When circulatory death doesn't come in time in potential organ donors

Angela Kotsopoulos[1]*, Nichon Jansen[2] and Wilson Farid Abdo[3]

[1] *Department of Intensive Care, Elisabeth-TweeSteden Hospital, Tilburg, the Netherlands, a.kotsopoulos@etz.nl*

[2] *The Dutch Transplant Foundation, Leiden, the Netherlands, n.jansen@transplantatiestichting.nl*

[3] *Department of Intensive Care Medicine, Radboud University Medical Center, Nijmegen, the Netherlands, f.abdo@radboudumc.nl*

* Correspondence: a.kotsopoulos@etz.nl

[1]Department of Intensive Care, Elisabeth-TweeSteden Hospital, Hilvarenbeekseweg 60, 5022 GC, Tilburg, the Netherlands,

Supplemental Table 1 Baseline characteristics of cDCD donors with a delayed time to death after withdrawal of life-sustaining therapy

Variable	Present cohort[a]	Suntharalingam et al. [a]	Wind et al.[a]	Xu et al. [a]	Brieva et al.[a]	Davila et al.[a]
Total number of patients included	804	191	211	219	159	417
Patients with a delayed circulatory death		83 (43%)[1]		53 (24.2%)[1]	78 (49%)[1]	117 (28%)[1]
	143 (18%)[2]	69 (36%)[2]	36 (17%)[2]	20 (9%)[2]		
Time to death median (range)	9.5 h (2h-5 days)	8 h (2h-3.3 days)	2h-3.8 days	2h-1.8 days		
Age (years)						
Median (Min-Max)	57 (26-74)	50 (16-62)	57 (29-75)		55 (17-65)	
Mean(SD)	55.6 (11.4)	48 (10.8)	55.4 (9.9)			51 (13)
Male No.(%)	84 (59%)	45 (65%)	27 (75%)		56 (52%)	76 (64.7%)
GCS						
Median (Min-Max)	4 (3-9)		3 (3-6)		5 (3-15)	
Mean (SD)	5.2 (2.7)		3.6 (0.6)			
Motor response absent or extensor	60 (49%)		25(69%)[3]			
Corneal reflex present				4 (20%)[4]		
Cough reflex present				9 (45%)[4]		57 (90.8%)[5]

Cause of death						
Medical[6]					60 (50.8%)	
Surgical[6]					19 (46.3%)	
PAE	49 (34%)	14 (20%)	17 (47%)			12 (10.3 %)
Cerebral**[7]**	78 (54%)	42 (61%)	18 (50%)	20 (100%)[8]		83 (70.9%)
Cerebral bleeding[9]	44 (29%)	27 (39%)	12 (33%)			
TBI	24 (17 %)	15 (22%)	3 (8%)			
ICH	33 (23%)		8 (22%)			
SAH	11 (6%)		4 (11%)			
CVA	10 (7%)		3 (8%)			
Cardiac						15 (12.8%)
Other	16 (12%)**[10]**	13 (19%)[11]	1 (3%)[12]			7 (6.3%)[13]

cDCD controlled donation after cardiac death, *CVA* cerebrovascular accident, *GCS* Glasgow Coma Scale, *ICH* intracranial hemorrhage, *Max* maximum, *Min* minimum, *PAE* post anoxic encephalopathy, *SAH* subarachnoid hemorrhage, *SD* standard deviation, *TBI* traumatic brain injury.

[a] Includes cDCD donors

[b] includes neurocritical patients

[1] Includes patients with a circulatory death after 60 minutes

[2] Includes patients with a circulatory death after 120 minutes

[3] Refers to patients with absent motor response

[4] Estimated from the DCD-N score (absent cough reflex equals two points and absent corneal reflex/motor response absent or extensor/oxygenation index >3 each one point) published by the authors

[5] The presence of a gag or cough reflex was added later and includes therefore a part of the population (2008-2009). Within accepted offers, 63 patients do not arrest within 60 minutes in a total of 178 patients

[6] The diagnosis on admission in not further specified

[7] Includes patients with a neurological condition or disorder

[8] Included only patients with traumatic brain injury and spontaneous cerebral hemorrhage

[9] Includes: intracranial hemorrhage (ICH) and subarachnoid hemorrhage (SAH)

[10] Includes: drug intoxication and pulmonary disorders

[11] Includes: cerebral infarction, cerebral edema, intracranial tumor or abscess, hydrocephalus, neurosurgical complication and liver failure

[12] Includes a patient after resection of pituitary adenoma

[13] Includes polytrauma, sepsis and other
